# Effect of cassava pulp treated with *Lactobacillus casei* TH14, urea, and molasses on gas kinetics, rumen fermentation, and degradability using the *in vitro* gas technique

**DOI:** 10.1016/j.heliyon.2024.e29973

**Published:** 2024-04-21

**Authors:** Sunisa Pongsub, Chaichana Suriyapha, Waewaree Boontiam, Anusorn Cherdthong

**Affiliations:** Tropical Feed Resources Research and Development Center (TROFREC), Department of Animal Science, Faculty of Agriculture, Khon Kaen University, Khon Kaen, 40002, Thailand

**Keywords:** Cassava pulp, Lactic acid bacteria, Additive, Ensiling, *In vitro* test

## Abstract

This study focused on examining the gas dynamics, rumen fermentation, and digestibility of ensiled cassava pulp (CSVP) using *Lactobacillus casei* TH14, urea, and molasses in the context of a laboratory experiment. All data in this study were analyzed using treatments arranged in 2 × 2 × 2 factorial arrangements using a completely randomized design. The *L.casei* TH14 additive (L) was factor A. Factor B was the molasses additive (M), while factor C was urea (U). There was no interaction effect of L, U, and M on gas production, volatile fatty acid (VFA) content, pH value, or ammonia-nitrogen level (*P<*0.05). The interaction of L, U, and M influenced *in vitro* dry matter digestibility (IVDMD) at 12 h (*P* < 0.05), and the CSVP fermented with the additions of L, U, and M together (LUM) was higher than the additions of CON, M, U, UM, and L on IVDMD (*P* < 0.05). However, the IVDMD values of adding LUM were higher in the control group (CON), M, U, UM, and L additive groups (*P* < 0.05). There was an interaction effect of L, U, and M on the protozoal count at 8 h (*P<*0.05), which had a lower protozoal count in the control group. In addition, acetic acid and butyric acid concentrations at 4 h and 8 h (*P<*0.05) were increased during the fermentation of CSVP using L and M combinations. Furthermore, the combination of U and M enhanced (*P<*0.05) average acetic acid, propionic acid, and pH at 4 h and 8 h while reducing (*P<*0.05) the gas generation from the insoluble portion (b). It was suggested that utilizing *L. casei* TH14 together with urea and molasses can enhance nutrient contents and improve the *in vitro* dry matter digestibility of CSVP, although it has no effect on ruminal fermentation or gas production.

## 1 Introduction

Currently, due to the limited availability of grazing space during the grazing season, livestock production is gradually increasing in order to feed caged cows freshly cut pasture on a daily basis; as a result, the need for adequate feed is an important issue [1]. Feedstuffs are necessary for total livestock productivity, especially in tropical locations during the summer in Thailand [2,3]. Furthermore, shortages have had a significant influence on production, both in terms of quality and quantity [4]. The application of industrial wastes and organic wastes from the food and crop industries, which still have some nutrients, as alternative animal feeds is an interesting global issue [5,6].

The cassava starch industry and cassava plants are widely farmed in the northeast of Thailand [7,8]. Cassava pulp (CSVP) is a residue of the starch manufacturing process, which could produce up to 500 tons of CSVP per day and might be a cause of pollution [9]. Whereas CSVP still contains some nutrients (60 % carbohydrates and 2.2 % crude protein), it may be fed to animals [[10], [11], [12]]. However, the use of CSVP was still limited due to its low protein content (2.17 % crude protein) and high fiber content, specifically cellulose at 20.65 % and lignin at 4.08 % [13]. Therefore, research has looked at using CSVP as animal feed by improving the quality of CSVP for animal diets, especially ruminants, using biological additions and other chemicals that have been identified and are acceptable for ruminants [[14], [15], [16]].

Various additives enhance the quality of fermented feed. Homofermentative lactic acid bacteria (LAB) shorten fermentation and prevent pathogenic microbes from producing organic acids [17]. In addition, the LAB addition in fermented feed improved *in vitro* digestibility and *in vivo* VFA production [18,19]. Furthermore, utilization of urea is the most typical nitrogen (N) source for microbial activity and breaking down plant cell walls [20]. Molasses serves as a carbon source for microbial fermentation, promotes lactic fermentation, and reduces undesirable fermentation due to its palatability [21,22]. As a means of stimulating fermentation, urea and molasses are often used as nitrogen and carbon supplies, respectively [23]. The production, development, and metabolism of microorganisms during acidogenesis fermentation require an optimal carbon-to-nitrogen (C/N) ratio [24]. Recent research by Pongsub et al. [10] indicated that CSVP fermented with *Lactobacillus casei* TH14 (*L. casei* TH14), urea, and molasses recovered DM, had fewer aerobic bacteria, and contained more LAB. In addition, the research recommended fermenting CSVP for 21 days since it produced a larger amounts of crude protein (CP) and lactic acid than other procedures [10]. While the existing research has provided valuable information on certain aspects of the fermentation process and its outcomes, a comprehensive understanding of the *in vitro* gas dynamics and related parameters is crucial for a more thorough evaluation of the efficacy and nutritional implications of this fermentation approach.

Therefore, the aim of this research was to investigate the influence of the CSVP fermented with *L. casei* TH14, urea, and molasses on gas kinetics, degradability, and fermentation characteristics using an *in vitro* gas technique. It was hypothesized that CSVP treated with a combination of *L. casei* TH14, molasses, and urea would be used as cattle feed to improve the efficiency of the ruminal fermentation process as well as ruminant growth performance.

## 2 Materials and methods

We confirmed that the research was conducted in accordance with Institutional Animal Care and Use Committee approval record No. IACUC-KKU-132/64 of Khon Kaen University and abides by all applicable regulations.

### 2.1 Experimental design and treatment

In this research, 2 × 2 × 2 factorial arrangements of treatments were used in a completely randomized design (CRD) with three replicates. Each factor came with or without additions. Factor A: lactic acid bacteria species *L. casei* strain TH14 at 0 and 2.5 milligram per kilogram of fresh matter (mg/kg FM). Factor B: molasses at 0 and 4 % DM of cassava pulp (CSVP). Factor C: urea at 0 and 4 % DM of CSVP. The levels of molasses and urea were obtained based on the recommendations of Norrapoke et al. [25].

### 2.2 Material and fermented feed preparation

The elements for the fresh CSVP were produced in Khon Kaen, in northeastern Thailand. The starter culture has 10^11^ cfu/g of *L. casei* TH14 [26] and was purchased from Bio Ag Khon Kaen Co., Ltd. (recommended usage rate: 2.5 mg/kg FM of material). Molasses and agricultural industrial-grade urea (46 % of nitrogen) were bought from a local feed store in Khon Kaen, Thailand. The L. *casei* TH14 was individually dissolved in sterilized distilled water. Urea and molasses were dissolved in sterilized distilled water together. Additives were sprayed into fresh cassava pulp. A vacuum sealer (Brother, Zhejiang Brother Packing Machinery Co., Ltd., Zhejiang, Wenzhou, China) was used to seal the 350 g of cassava pulp per bag that was packed into the bag. There were three replicates of cassava pulp ensiled with each additive type. After that, it was kept at ambient temperature for 21 days of ensiling.

The fermented CSVP samples were subjected to heat drying at a temperature of 60 °C. After that, they were milled into particles using a colander with a mesh size of 1 mm. The chemical compositions of the samples were evaluated using the standard method of the Association of Official Analytical Chemists (AOAC), which included the determination of dry matter (DM), organic matter (OM), crude protein (CP), and ether extract (EE) [27,28]. The Ankom fiber analyzer (ANKOM 200, ANKOM Technology, New York, NY, USA) was used to assess the neutral detergent fiber (NDF) and acid detergent fiber (ADF) [29]. The chemical compositions of CSVP were altered with various additions, as described in Table 1.

**Table 1:** The chemical compositions of cassava pulp were treated with different additives.

Item^1^	CON	M	U	UM	L	LM	LU	LUM
Dry matter (%)	16.34	17.17	17.78	17.95	16.28	17.01	16.96	17.61
	(% DM)							
Organic matter	92.67	92.83	92.86	92.56	92.37	92.58	92.87	92.55
Crude protein	2.78	2.99	15.41	13.47	2.95	3.08	15.40	14.05
Ether extract	0.48	0.50	0.32	0.22	0.46	0.37	0.28	0.20
Neutral detergent fiber	36.90	33.02	33.30	31.91	35.14	33.57	35.19	31.83
Acid detergent fiber	22.81	20.19	21.41	21.06	22.70	21.17	21.46	20.87

CON = no additive cassava pulp; M = cassava pulp fermented with molasses; U = cassava pulp fermented with urea; UM = cassava pulp fermented with urea and molasses; L = cassava pulp fermented with *Lactobacillus casei* TH14; LM = cassava pulp fermented with *Lactobacillus casei* TH14 and molasses; LU = cassava pulp fermented with *Lactobacillus casei* TH14 and urea; LUM = cassava pulp fermented with *Lactobacillus casei* TH14, urea, and molasses.

### 2.3 Substances and incubation

Rumen fluids were taken from two cows with a body weight of 450 ± 35 kg by cannulating them. The dairy bulls of crossbred Holstein Friesian in cages had access to a total mix ratio (TMR) diet, which included 14 % of CP and 75 % total digestible nutrients (TDN), with 10 % refusals permitted, and a new feeding was served at 8.00 in the morning and 4.00 in the afternoon. Mineral blocks and water were options. In advance of collecting the ruminal fluid, the host animals received the diet for 21 days. Before morning feeding from the host cows, 1200 mL of rumen fluid was meticulously collected, sieved through four layers of cheesecloth, and delivered to the lab in 39 °C bottles. The whole procedure was anaerobic and sterile. An *in vitro* vial with a capacity of 50 mL was filled with 0.5 g of ensiled cassava pulp samples that were pulverized and evenly distributed. The artificial saliva was prepared following the procedure described by Menke et al. (1979). In addition, it was mixed well with the rumen fluid in an environment without oxygen and applied to modulate the pH in the rumen control system. Sanitizing the vials and delivering 40 mL of the ruminal fluid solution combination was accomplished by using a 60-mL syringe equipped with a 1.5 mm 20-gauge needle. This was done in order to prevent oxygen from entering the vials. All bottles containing the various substrate treatments were placed in a 39 °C thermal oven.

### 2.4 Samples and analyses

The ruminal pH was measured regularly via a pH meter at 4 and 8 h (incubate separate sets of vials), ammonia nitrogen (NH_3_–N), and volatile fatty acid (VFA) values were measured, and the measurement of rumen fluid acidity or basicity (pH value) was done via a pH meter. In accordance with the methodology proposed by Fawcett and Scott [30], a spectrophotometer was utilized in order to determine the concentration of NH_3_–N. Volatile fatty acid evaluation using gas chromatography equipped with the Porter and Murray [31] method. Separate sets of bottles were incubated for 12 and 24 h, respectively, after which the sample was filtered through pre-weighed Gooch crucibles with a porosity of 40 mm. The DM residue was then left to dry in a hot air oven at 100 °C for 24 h. In order to evaluate the *in vitro* DM digestibility (IVDMD) and the *in vitro* OM digestibility (IVOMD), the DM residue was weighed. The residuals that had dried out were subjected to a 3-h electric burner burn at 550 °C [32]. The direct microbiological counting was performed by a method developed by Galyean [33]. Using the model developed by Sommart et al. [34] as a guidance, the following equation was used to calculate the gas production kinetics at 1, 2, 4, 6, 12, 18, 24, 48, 72, and 96 h after incubation:

$$Y=a+b\;\left(1\;–\;e^{-\text{ct}}\;\right)$$

where “a” is the intercept, which ideally captures the fermentation of the sample extract, “b” is the fermentation of the amount of excess (which is time fermentable), “c” is the degree of gas production, “|a| + b” is the possible extent of gas production, and “Y” is the gas produced at period “t”.

### 2.5 Statistical determination

All experiment data were statistically analyzed as 2 × 2 × 2 factorial arrangements of treatments were used in a completely randomized design (CRD) using SAS's GLM method (Version 9.0; SAS Institute Inc., Cary, NC, U.S.A). The following model was used to analyze the data:

$$Y_{ijk}=\mu +\alpha_{i}+\beta_{j}+\gamma_{k}+{\alpha \beta }_{ij}+{\alpha \gamma }_{ik}+{\beta \gamma }_{jk}+{\alpha \beta \gamma }_{ijk}+\varepsilon_{ijk}$$

Where *Y*_*ijk*_ = Observation values; μ = Overall mean; *α*_*i*_ = Effect of main factor A (dose of LAB at *i* when *i* = 1 to 2); *β*_*j*_ = Effect of main factor B (dose of urea at *j* when *j* = 1 to 2); *γ*_*k*_ = Effect of main factor C (dose of molasses at *k* when *k* = 1 to 2); *αβ*_*ij*_ = Interaction of A and B at *ij*; *αγ*_*ik*_ = Interaction of A and C at *ik*; *βγ*_*jk*_ = Interaction of B and C at *jk*; *αβγ*_*ijk*_ = Interaction of A, B, and C at *ijk*; *ε*_*ijk*_ = Error term. Each treatment had three replications. At a significance level of P < 0.05, which is recognized as a statistically significant difference, the means were compared using Duncan's new multiple-range tests [35].

## 3 Result

### 3.1 Kinetics of gas

Table 2 shows the effects of fermented CSVP with varied additions on gas production kinetics and gas accumulation *in vitro* after 96 h of incubation. As a consequence of the fermentation of CSVP with L × U × M, gas production occurred immediately from the soluble fractions (a), indicating that the insoluble fractions (c) had no impact on each other (P > 0.05). Combining urea and molasses reduced the amount of gas produced from the insoluble fraction (b) and the cumulative gas volume after 96 h (P < 0.05). Additionally, the total potential for gas production (|a|+b) was 6.90 % lower in the urea group compared to the non-urea group, and this difference was statistically significant (P < 0.01).

**Table 2:** *In vitro* kinetics and accumulation of gas after 96 h of incubation affected by fermented cassava pulp with different additives.

Items^a^		a	b	c	|a|+b	Gas accumulation (ml/0.5 g DM substrate)
CON		−10.11	150.84	0.07	160.96	137.03
M		−10.40	143.31	0.07	153.71	126.97
U		−9.63	134.58	0.07	144.21	120.20
UM		−10.32	137.43	0.07	147.75	126.13
L		−7.67	149.97	0.07	157.64	137.73
LM		−8.42	144.96	0.07	153.38	131.00
LU		−11.09	133.63	0.07	144.72	121.67
LUM		−10.61	135.25	0.07	145.86	125.27
SEM		1.36	2.34	0.0001	2.81	3.63
Orthogonal contrasts						
L	no	−9.45	141.54	0.07	151.66	127.58
	yes	−10.12	140.95	0.07	150.40	128.92
U	no	−9.15	147.27^a^	0.07	156.42^a^	133.18^a^
	yes	−10.41	135.22^b^	0.07	145.63^b^	123.32^b^
M	no	−9.62	142.26	0.07	151.88	129.16
	yes	−9.94	140.24	0.07	150.18	127.34
Interaction effect						
L × U		0.13	0.56	0.31	0.78	0.69
L × M		0.86	0.85	0.11	0.94	0.92
U × M		0.83	0.02	0.55	0.06	0.02
L × U × M		0.68	0.58	0.91	0.51	0.59

^a-b^ Means within columns with difference superscript differ at *P<*0.05; SEM = standard error of mean.

a CON = no additive cassava pulp; M = cassava pulp fermented with molasses; U = cassava pulp fermented with urea; UM = cassava pulp fermented with urea and molasses; L = cassava pulp fermented with *Lactobacillus casei* TH14; LM = cassava pulp fermented with *Lactobacillus casei* TH14 and molasses; LU = cassava pulp fermented with *Lactobacillus casei* TH14 and urea; LUM = cassava pulp fermented with *Lactobacillus casei* TH14, urea, and molasses. A = the intercept, which ideally reflects the fermentation of the soluble fraction, b = the fermentation of the insoluble fraction (which is with the time fermentable), c = is the rate of gas production, |a| + b = the potential extent of gas production.

### 3.2 *In vitro* digestibility

Table 3 illustrates the *in vitro* digestibility of dry matter (IVDMD) and organic matter (IVOMD) of fermented CSVP with various additions after 12 and 24 h of incubation. There was an interaction impact of L, U, and M (P < 0.05) in the IVDMD at 12 h after incubation. Compared to the CON group, the IVDMD of the CSVP fermented with LUM was 22.89 % higher at 12 h (P < 0.05). Other digestibility measures, however, show no interaction or main factor influence (P > 0.05).

**Table 3:** *In vitro* digestibility of dry matter (IVDMD) and organic matter (IVOMD) of fermented cassava pulp with different additives after incubation at 12 and 24 h.

Items^a^		IVDMD (% DM)			IVOMD (% DM)		
		12 h	24 h	Mean	12 h	24 h	Mean
CON		36.83^c^	53.95	45.39	48.12	61.38	54.75
M		33.06^de^	52.29	42.67	47.34	61.39	54.37
U		30.71^e^	57.74	44.22	48.29	64.32	56.30
UM		35.52^cd^	54.84	45.18	46.29	63.97	55.13
L		41.19^b^	53.10	47.14	47.59	60.07	53.83
LM		43.36^ab^	54.46	48.91	49.81	60.69	55.25
LU		42.50^ab^	54.69	48.60	49.45	60.29	54.87
LUM		45.26^a^	51.61	48.43	51.39	57.62	54.51
SEM		0.72	1.57	3.95	1.40	1.41	0.93
Orthogonal contrasts							
L	no	34.50^b^	54.60	44.37	47.51	62.79	55.14
	yes	43.08^a^	53.29	48.27	49.56	59.58	54.61
U	no	38.61	53.62	46.03	48.22	60.81	54.55
	yes	39.61	54.22	46.61	48.86	61.36	55.20
M	no	38.82	54.42	46.34	48.36	61.69	54.94
	yes	39.30	53.44	46.30	48.71	60.33	54.81
Interaction effect							
L ˣ U		0.08	0.29	0.98	0.40	0.15	0.55
L ˣ M		0.07	0.58	0.81	0.19	0.86	0.44
U ˣ M		0.02	0.39	0.90	0.77	0.52	0.45
L ˣ U ˣ M		0.02	0.64	0.69	0.85	0.63	0.76

^a-e^ Means within columns with difference superscript differ at *P<*0.05; SEM = standard error of mean.

a CON = no additive cassava pulp; M = cassava pulp fermented with molasses; U = cassava pulp fermented with urea; UM = cassava pulp fermented with urea and molasses; L = cassava pulp fermented with *Lactobacillus casei* TH14; LM = cassava pulp fermented with *Lactobacillus casei* TH14 and molasses; LU = cassava pulp fermented with *Lactobacillus casei* TH14 and urea; LUM = cassava pulp fermented with *Lactobacillus casei* TH14, urea, and molasses.

### 3.3 Concentration of volatile fatty acid in in vitro

At both the 4 and 8 h incubation points, Table 4 displays the effects on VFAs of fermented CSVP with different additions. Interactions between L, U, and M did not affect total VFA, acetic acid (C2), propionic acid (C3), or butyric acid (C4). The interaction between U and M, on the other hand, had an influence on the average incubation periods for the mean values of C2 and C3 (P < 0.05). In comparison to other treatments, the interaction of U and M had a lower amount of C2 values and higher C3 values for the average of the incubation durations. After 8 h of incubation, the L and M affected the percentages of C2, C3, and C4 and the average concentration (P < 0.05). The average percentage of C2 values was lower, while the C3 and total VFA values were greater. The combination of L and U had an impact on the percentages of C2, C4, and total VFA after 8 h of incubation (P < 0.05), with a lower average percentage of C4 values and greater C2 and total VFA values. Additionally, the percentage of C2 and total VFA were found in higher concentrations in the urea-influenced group compared to the non-urea group as a result of the main influence of urea. As compared to the group that did not use urea, the urea group had decreased C3 and C4 concentrations.

**Table 4:** *In vitro* volatile fatty acid profiles (VFAs) influenced by fermented cassava pulp with different additives after incubation at 4 and 8 h.

Items^a^		Acetic acid (% total VFA)			Propionic acid (% total VFA)					Butyric acid (% total VFA)			Total VFA (mmol/L)					
		4h	8h	Mean	4h		8h	Mean		4h	8h	Mean	4h		8h		Mean	
CON		72.35	72.10	72.23	17.07		17.72	17.39		10.58	10.18	10.38	40.4		55.5		47.9	
M		73.31	73.58	73.45	16.53		17.06	16.80		10.16	9.36	9.76	42.6		64.0		53.3	
U		75.31	71.92	73.62	15.58		18.31	16.95		9.11	9.77	9.44	46.2		57.6		51.9	
UM		74.50	72.00	73.25	16.12		18.13	17.13		9.38	9.87	9.63	45.4		58.3		51.8	
L		74.07	73.66	73.87	16.25		16.87	16.56		9.68	9.47	9.57	44.6		58.6		51.6	
LM		74.06	72.46	73.26	16.11		17.50	16.80		9.83	10.04	9.94	43.7		58.2		51.0	
LU		75.26	74.03	74.64	15.96		17.33	16.64		8.78	8.64	8.71	49.0		67.0		58.0	
LUM		74.47	72.61	73.54	16.26		18.24	17.25		9.27	9.15	9.21	45.5		64.3		54.9	
SEM		0.67	0.29	0.21	0.35		0.19	0.11		0.33	0.19	0.13	1.50		1.91		0.99	
Orthogonal contrasts rowhead																		
L	no	73.87	72.40^b^	73.13^b^		16.32	17.81^a^	17.07^a^	9.81		9.79^a^	9.80^a^		43.66		58.85^b^		51.25^b^
	yes	74.47	73.19^a^	73.83^a^		16.15	17.48^b^	16.81^b^	9.39		9.33^b^	9.36^b^		45.73		62.03^a^		53.88^a^
U	no	73.45^b^	72.95	73.20^b^		16.49	17.29^b^	16.89	10.06^a^		9.76^a^	9.91^a^		42.86^b^		59.08		50.97^b^
	yes	74.88^a^	72.64	73.76^a^		15.98	18.00^a^	16.99	9.14^b^		9.36^b^	9.25^b^		46.52^a^		61.80		54.16^a^
M	no	74.25	72.93	73.59		16.21	17.56	16.89	9.54		9.51	9.53		45.05		59.67		52.36
	yes	74.09	72.66	73.37		16.25	17.73	16.99	9.66		9.61	9.63		44.33		61.21		52.77
Interaction effect rowhead																		
L × U		0.22	0.02	0.87		0.11	0.41	0.13	0.42		0.01	0.28		0.59		0.01		0.05
L × M		0.64	<0.01	0.01		0.88	<0.01	0.01	0.43		0.01	0.02		0.21		0.05		0.03
U × M		0.22	0.08	0.02		0.16	0.20	0.02	0.31		0.14	0.07		0.23		0.09		0.05
L × U × M		0.62	0.19	0.18		0.54	0.74	0.32	0.71		0.10	0.17		0.91		0.34		0.41

^a-b^ Means within columns with difference superscript differ at *P<*0.05; SEM = standard error of mean.

a CON = no additive cassava pulp; M = cassava pulp fermented with molasses; U = cassava pulp fermented with urea; UM = cassava pulp fermented with urea and molasses; L = cassava pulp fermented with *Lactobacillus casei* TH14; LM = cassava pulp fermented with *Lactobacillus casei* TH14 and molasses; LU = cassava pulp fermented with *Lactobacillus casei* TH14 and urea; LUM = cassava pulp fermented with *Lactobacillus casei* TH14, urea, and molasses.

### 3.4 Concentration of ruminal NH_3_–N and pH

The effects on ruminal pH and NH_3_–N of fermented CSVP with different additions are shown in Table 5. The interaction of L, U, and M showed no influence on pH and NH_3_–N levels after 4 and 8 h of incubation, as well as the mean value (P > 0.05). The interaction between U and M, on the other hand, had an influence on the pH at 8 h after incubation and the average value (P < 0.05), which is substantially similar to other treatments in terms of value. Urea had a significant effect on NH_3_–N at 4, 8, and average (P < 0.01). Furthermore, the primary impact of molasses releasing NH_3_–N at 4 h and the average value (P < 0.05).

**Table 5:** Effect of cassava pulp fermented with different additives on ruminal pH and ammonia nitrogen.

Items^a^		pH			Ammonia-N (mg/dl)		
		4h	8h	Mean	4h	8h	Mean
CON		7.10	6.91	7.00	24.97	21.22	23.09
M		7.05	6.89	6.97	24.20	17.65	20.93
U		7.03	6.87	6.95	29.42	29.33	29.38
UM		7.00	6.96	6.98	29.39	28.74	29.06
L		7.05	6.94	6.99	25.22	20.01	22.62
LM		7.01	6.79	6.90	21.07	20.41	20.74
LU		6.89	6.81	6.85	29.41	28.21	28.81
LUM		6.95	6.91	6.93	29.19	26.96	28.07
SEM		0.05	0.04	0.03	0.62	1.12	0.46
Orthogonal contrasts							
L	no	7.04	6.91	6.97^a^	26.99	24.23	25.61
	yes	6.97	6.86	6.92^b^	26.99	23.90	25.06
U	no	7.05	6.88	6.96	23.87^b^	19.82^b^	21.84^b^
	yes	6.97	6.89	6.93	29.35^a^	28.31^a^	28.83^a^
M	no	7.02	6.88	6.95	27.25^a^	24.69	25.97^a^
	yes	7.00	6.89	6.94	25.96^b^	23.44	24.70^b^
Interaction effect							
L × U		0.55	0.77	0.43	0.25	0.28	0.59
L × M		0.55	0.46	0.93	0.13	0.42	0.94
U × M		0.47	0.04	0.03	0.06	0.74	0.10
L × U × M		0.63	0.36	0.24	0.17	0.27	0.67

^a-b^ Means within columns with difference superscript differ at *P<*0.05; SEM = standard error of mean.

a CON = no additive cassava pulp; M = cassava pulp fermented with molasses; U = cassava pulp fermented with urea; UM = cassava pulp fermented with urea and molasses; L = cassava pulp fermented with *Lactobacillus casei* TH14; LM = cassava pulp fermented with *Lactobacillus casei* TH14 and molasses; LU = cassava pulp fermented with *Lactobacillus casei* TH14 and urea; LUM = cassava pulp fermented with *Lactobacillus casei* TH14, urea, and molasses.

### 3.5 Microbial concentrations

Table 6 demonstrates the microbial communities altered by fermented CSVP with various additions after 4 and 8 h of incubation. When compared to other groups, CON and L had a reduced protozoal count at 8 h, which is influenced by an interaction effect of U, M, and L (P < 0.05). The mean protozoal number was affected by the interaction between U and M (P < 0.05). There are no changes in the bacterial population at 4 and 8 h (P > 0.05), however, the major impact of molasses affects the mean bacterial count by 0.36 % (P < 0.05). In addition, dietary interventions had no effect on fungus counts (P > 0.05).

**Table 6:** Microbial populations after incubation at 4h and 8h influenced by fermented cassava pulp with different additives.

Items^a^		Bacteria			Protozoa			Fungi		
		(Log10 cell/ml)								
		4h	8h	Mean	4h	8h	Mean	4h	8h	Mean
CON		8.27	8.40	8.34	5.15	4.70^c^	4.93	5.30	5.30	5.30
M		8.37	8.47	8.44	5.15	5.39^a^	5.27	5.24	5.35	5.30
U		8.29	8.43	8.36	5.15	4.70^c^	4.93	5.30	5.30	5.30
UM		8.31	8.48	8.40	5.15	5.15^ab^	5.15	5.15	5.30	5.23
L		8.30	8.45	8.38	5.48	4.70^c^	5.09	5.24	5.15	5.20
LM		8.38	8.48	8.43	5.15	5.39^a^	5.27	5.15	5.30	5.23
LU		8.32	8.45	8.38	5.30	5.24^a^	5.27	5.15	5.24	5.20
LUM		8.30	8.43	8.37	5.15	5.00^b^	5.08	5.30	5.30	5.30
SEM		0.03	0.02	0.02	0.11	0.08	0.05	0.08	0.05	0.05
Orthogonal contrasts										
L	no	8.31	8.44	8.38	5.15	5.03	5.07^b^	5.25	5.31	5.28
	yes	8.32	8.45	8.39	5.27	5.08	5.18^a^	5.21	5.25	5.23
U	no	8.33	8.45	8.39	5.23	5.04	5.14	5.23	5.29	5.25
	yes	8.30	8.45	8.37	5.19	5.07	5.105	5.23	5.28	5.26
M	no	8.29	8.43	8.36^b^	5.27	4.85^b^	5.05^b^	5.25	5.25	5.25
	yes	8.34	8.46	8.41^a^	5.15	5.23^a^	5.19^a^	5.21	5.31	5.26
Interaction effect										
L × U		0.77	0.28	0.56	0.64	0.33	0.58	0.60	0.43	0.41
L × M		0.53	0.15	0.16	0.23	0.01	0.58	0.35	0.37	0.23
U × M		0.07	0.42	0.08	0.64	<0.01	0.03	0.60	0.43	0.98
L × U × M		0.86	0.70	0.88	0.64	0.02	0.20	0.26	0.82	0.41

^a-b^ Means within columns with difference superscript differ at *P<*0.05; SEM = standard error of mean.

a CON = no additive cassava pulp; M = cassava pulp fermented with molasses; U = cassava pulp fermented with urea; UM = cassava pulp fermented with urea and molasses; L = cassava pulp fermented with *Lactobacillus casei* TH14; LM = cassava pulp fermented with *Lactobacillus casei* TH14 and molasses; LU = cassava pulp fermented with *Lactobacillus casei* TH14 and urea; LUM = cassava pulp fermented with *Lactobacillus casei* TH14, urea, and molasses.

## 4 Discussions

### 4.1 Kinetics of gas

**Fig. 1:**
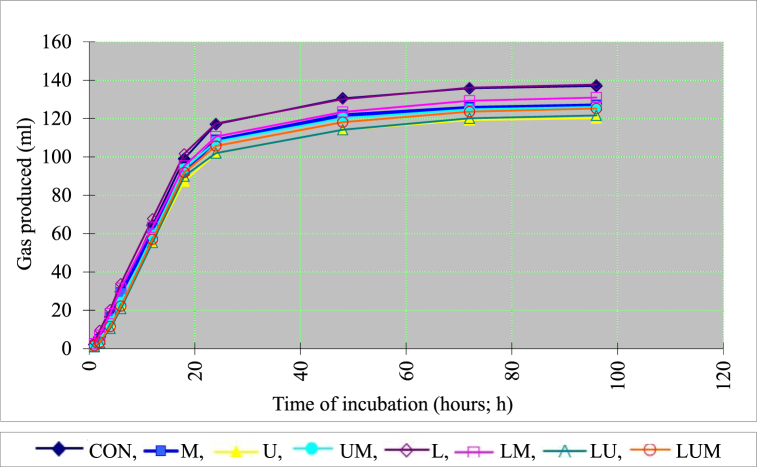
Gas accumulation curves of cassava pulp fermented with different additives throughout incubation times (0–96 h). CON = no additive cassava pulp; M = cassava pulp fermented with molasses; U = cassava pulp fermented with urea; UM = cassava pulp fermented with urea and molasses; L = cassava pulp fermented with *Lactobacillus casei* TH14; LM = cassava pulp fermented with *Lactobacillus casei* TH14 and molasses; LU = cassava pulp fermented with *Lactobacillus casei* TH14 and urea; LUM = cassava pulp fermented with *Lactobacillus casei* TH14, urea, and molasses.

Gas production kinetics were predicated on the relative ratios of soluble and insoluble portions rather than those of degradable and undegradable particles as in feed [36]. Gas production optimally reflects soluble portion fermentation (a) in this study, revealing a negative value. This was likely owing to a variation in the exponential source of fermentation or microbial colonization belatedly [37]. Hence, prior studies found that the absolute amount of a, (|a|), might denote the ideal digestion of the solubility part [4,37]. Moreover, gas generation from the (a) fraction did not change with dietary treatment. This is most likely related to the equality of structural and soluble carbohydrates in agro-industrial residues [38]. Furthermore, changes in the CP of the fermented CSVP affect the amount of gas produced [4,37]. Nevertheless, Chanthakhoun and Wanapat [39] found no differences in the gas (a) of ten feedstuffs that consist of crop residues and roughage. This study indicated that the insoluble fraction of gas (b), |a|+b, and the cumulative gas decreased with the factor of urea addition in CSVP fermentation. The effect of CSVP with different additives, as determined by *in vitro* rumen simulation, on gas cumulation is shown in Fig. 1. Similar to previous studies, this one demonstrated that the use of urea with molasses or even the addition of urea alone as a supplement in fermented feed indicates in a decline in the gas synthesis of the fermentation [40,41]. The efficiency of gas production in any measurement (|a|+b) and gas accumulation decreased. Protein breakdown releases ammonia, which neutralizes hydrogen ions from VFA without releasing carbon dioxide. This means that feedstuffs with substantial CP generate a small amount of gas during digestion, regardless of how fast they break down [42]. The following are the key issues that need to be clarified about the research, which found that the fermentation of casein created only 32 % gas in comparison to the fermentation of carbohydrates [43].

### 4.2 In vitro digestibility

The present study revealed that an interaction with *Lactobacillus casei* TH14, urea, and molasses caused a greater incidence of IVDMD at 12 h in LUM. Lactic acid bacteria improve cellulose digestibility by producing the enzyme ferulate esterase, which converts ferulic acid to arabinoxylan [44]. In addition, ferulate esterase is found in several Lactobacillus subspecies [45]. Arabinoxylan helps fibrous-digesting enzymes through the cell wall's lignin-cellulose [46]. Additionally, adding urea to CSVP as an alkaline agent produced ammonium hydroxide (NH_4_OH). This might lead to the expansion of the hemicellulose-lignin complex inside CSVP, which in turn would increase fiber digestion by chemically dissolving its ester linkages [4,47,48]. Moreover, urea is a typical nitrogen nutrient and an essential component of proteins, which are required for microbial activity [49,50]. In this study, molasses serves as a carbon source of energy for bacteria. Carbon is needed by microbes for both energy production and mass formation; for example, lactic acid bacteria use carbon elements for energy 70 % of the time during development and 30 % during metabolic operations [49,50]. The addition of molasses to the fermentation process enhanced the decomposition of the plant fibers, as shown by Rezaei et al. [51] who found that the NDF and ADF in fermented feed decreased as molasses levels increased. Therefore, it might be claimed that LAB, urea, and molasses have a beneficial effect by decreasing fiber and increasing digestibility.

### 4.3 Concentration of volatile fatty acid in in vitro

The current study found that the total VFA at 8 h and the mean content value are influenced by the interaction of L × U and L × M. This is probably due to the addition of *L. casei* TH14 during the ensiling process that generates lactic acid, which could degrade structural carbohydrates into smaller molecules by acid hydrolysis at low pH [52]. Furthermore, the LAB generates lactic acid through a biological conversion of soluble carbohydrates (molasses), and the addition of urea increases microbial activity [49,[53], [54], [55]]. Consequently, to feed the microbes in the rumen the carbs that they need to rapidly convert to pyruvate, which they will use to make acetate (C2), propionate (C3), and butyrate (C4) [56]. Similar to Lee et al. [57], who discovered that whole-crop rice (WCR)-inoculated LAB had higher total VFA and C2 concentrations but lower C3 concentrations than control fermented feed. Because WCR fermentation is mostly structural carbohydrates, a high C2 content indicates strong rumen digestion. High rumen C2 may lower C3. Moreover, the addition of urea as an essential nitrogen source increased the action of rumen microorganisms in digesting carbohydrates and increased bacterial end-product (VFA) activity in ruminal fluid [49,58]. However, urea depleted C4 in this experiment, contrary to previous findings that urea had no effect on C4 [47,58]. This variation may be due to variations in C2 and C3 concentrations, which may affect the C4 concentration ratio [59]. Whereas molasses had no direct influence in this experiment, it had an impact in combination with other factors. Based on their research, So et al. [26] showed that adding molasses to the ensiling stage increased the nutritional value, LAB population, lactic acid, organic acid, and ruminal VFA concentrations in agricultural by-products [19,26].

### 4.4 Concentration of ruminal NH_3_–N and pH

The optimal pH for cellulose digestion appears to differ based on material and/or rumen fluid supply [60]. The ruminal *in vitro* pH values in the present work were close to the optimum values of 6.89–7.10, whereas they have typically been performed in the range of 6.4–6.9 in several studies that grass-fed ruminants often have a physiological ruminal pH of 6–7 [61]. In this study, the interaction impact of LAB, urea, and molasses had no influence on pH value. Nevertheless, the pH was decreased by LAB-fermented CSVP. The pH of fermented feed that was inoculated with LAB was shown to be decreased, according to Oliveira et al. [62] and Zhao et al. [63]. Lactate and lactic acid that are generated from LAB lead to a reduction in ruminal pH when fed rapidly fermentable carbohydrates [64].

Our results show that the rapid breakdown of urea into ruminal NH_3_–N by enzymes produced by bacteria is acceptable, as the addition of urea boosted the NH_3_–N content [65]. Research by Araba et al. [66], which looked at the impact of using sugar beet molasses instead of barley, found that the NH_3_–N content dropped because of the molasses. Similarly, Shotorkhoft et al. [67] noticed a drop in ruminal NH_3_–N content due to the extraordinarily high levels of molasses in the diets of sheep. This may result in a greater incorporation of NH_3_–N and fermentable energy into microbial nitrogen as a precursor for microbial protein synthesis. Optimal NH_3_–N values between 15 and 30 mg/100 mL that sustain protein metabolism in the rumen might promote the growth of microbial protein synthesis (MPS) [68,69]. In this experiment, the appropriate NH_3_–N concentration achieved by adding molasses may be advantageous to MPS.

### 4.5 Microbial concentrations

In the present investigation, the protozoal counts changed after 8 h of incubation with interaction variables. The key factors affecting MPS, according to Ezequias [70], are the accessibility of carbs, the presence of ruminal digestible protein, and the ideal pH of the rumen. The essential precursors of protozoal metabolism are starch and sugar in high-grain diets [71,72]. Furthermore, Nile et al. [73] noted an increase in overall protozoal numbers, which may be explained by the common value of rumen pH, the energy that can be utilized by them (in the form of SCFA synthesis), and the nitrogen (in the form of adequate NH_3_–N concentration) for greater MPS. Furthermore, *Entodiniomorphid* protozoa were assumed to utilize fiber at a lower rate than starch or soluble sugar in molasses, resulting in enhanced fractional growth rates, particularly in species that are poor in cellulase and/or hemicellulase activities [74]. Moreover, the LAB inoculant was evaluated to see whether it might increase fermented feed digestibility by producing ferulate esterase, which breaks hemicellulose-lignin connections to make fiber more digestible [75]. Consequently, in this research, the interaction of lactic bacteria, urea, and molasses may benefit substances that promote protozoal growth. Therefore, maximizing the usage of cassava pulp is one area where the use of additives like *L. casei* TH14, urea, and molasses might have practical significance for livestock nutrition. Farmers and animal nutritionists may consider incorporating this combination to improve the nutritional value of feed, potentially leading to better animal performance.

## 5 Conclusions and perspectives

The CSVP can improve the nutritional values through a fermentation process with different additives. The combination of urea and molasses in CSVP ensiling led to an enhance in acetic acid, propionic acid, and pH while decreasing the gas production from the insoluble fraction (b). The quantities of acetic and butyric acids are enhanced during the CSVP fermentation process when *L. casei* TH14 and molasses are used. At 12 h, *L. casei* TH14 combined with urea and molasses reduces IVDMD more effectively. According to the findings of this study, it was recommended that the addition of *L. casei* TH14, urea, and molasses could be one approach for nutrient quality improvement in CSVP and might be potentially used as ruminant feed. Nevertheless, more *in vivo* studies are needed to determine the efficacy and impact of the CSVP fermented for animal feed. This additional research will provide a more comprehensive understanding of the effectiveness of the proposed additives in a real-world ruminant feeding scenario.

## 6 Ethics statement

The authors certify that the ethical standards of the journal, which are detailed on the author requirements page, have been followed and that the necessary ethical review committee permission has been obtained. The authors swear that they followed EU rules about how to protect animals used in research.

## Funding statement

The authors would like to extend their deepest appreciation to the National Science, Research and Innovation Fund (NSRF) for supporting Khon Kaen University's Fundamental Fund. Also recognized was the Research Program on the Research and Development of Winged Bean Root Utilization as Ruminant Feed, Increase Production Efficiency and Meat Quality of Native Beef and Buffalo Research Group and Research and Graduate Studies, Khon Kaen University (KKU). 10.13039/100022395Graduate School of KKU's Research Fund for Supporting Lecturers to Admit High Potential Students to Study and Research on His Expert Program awarded Miss Sunisa Pongsub a grant (Grant no. 631JT103).

## Data availability statement

Data will be made available on request.

## CRediT authorship contribution statement

**Sunisa Pongsub:** Writing – review & editing, Writing – original draft, Validation, Methodology, Investigation, Formal analysis, Data curation, Conceptualization. **Chaichana Suriyapha:** Writing – review & editing, Writing – original draft, Validation, Investigation. **Waewaree Boontiam:** Writing – original draft, Supervision, Resources, Funding acquisition. **Anusorn Cherdthong:** Writing – review & editing, Writing – original draft, Visualization, Validation, Supervision, Resources, Project administration, Funding acquisition, Formal analysis, Conceptualization.

## Declaration of competing interest

The authors declare that they have no known competing financial interests or personal relationships that could have appeared to influence the work reported in this paper.
